# Supplementary material for: Sex bias determines MERS-CoV infection outcomes in a mouse model of differential pathogenicity
Source: bioRxiv. 2025 Jun 19:2025.06.19.660369. Preprint. [Version 1] doi: 10.1101/2025.06.19.660369 (PMC12224536; doi:10.1101/2025.06.19.660369)
Supplement: 1 [file NIHPP2025.06.19.660369V1-supplement-1.pdf]

bioRxiv preprint doi: https://doi.org/10.1101/2025.06.19.660369; this version posted June 19, 2025. The copyright holder for this preprint (which was not certified by peer review) is the author/funder. This article is a US Government work. It is not subject to copyright under 17 USC 105 and is also made available for use under a CC0 license.

Supplementary Figures

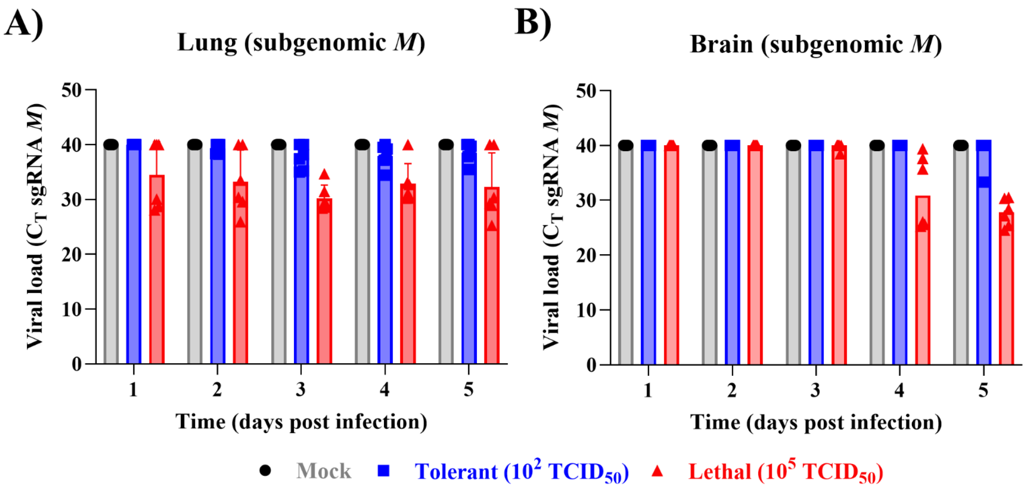

Figure S1. Mice infected with a high dose of MERS-CoV experience greater subgenomic replication in the lungs and brain compared to low dose mice. Lung (**A**) and brain (**B**) tissues were collected days 1-5 post-infection and RNA was extracted to assess viral load by RT-qPCR. The MERS-CoV subgenomic (sgRNA) *M* gene was used to assess subgenomic replication. Data represent the median for 6 mice per group.

bioRxiv preprint doi: https://doi.org/10.1101/2025.06.19.660369; this version posted June 19, 2025. The copyright holder for this preprint (which was not certified by peer review) is the author/funder. This article is a US Government work. It is not subject to copyright under 17 USC 105 and is also made available for use under a CC0 license.

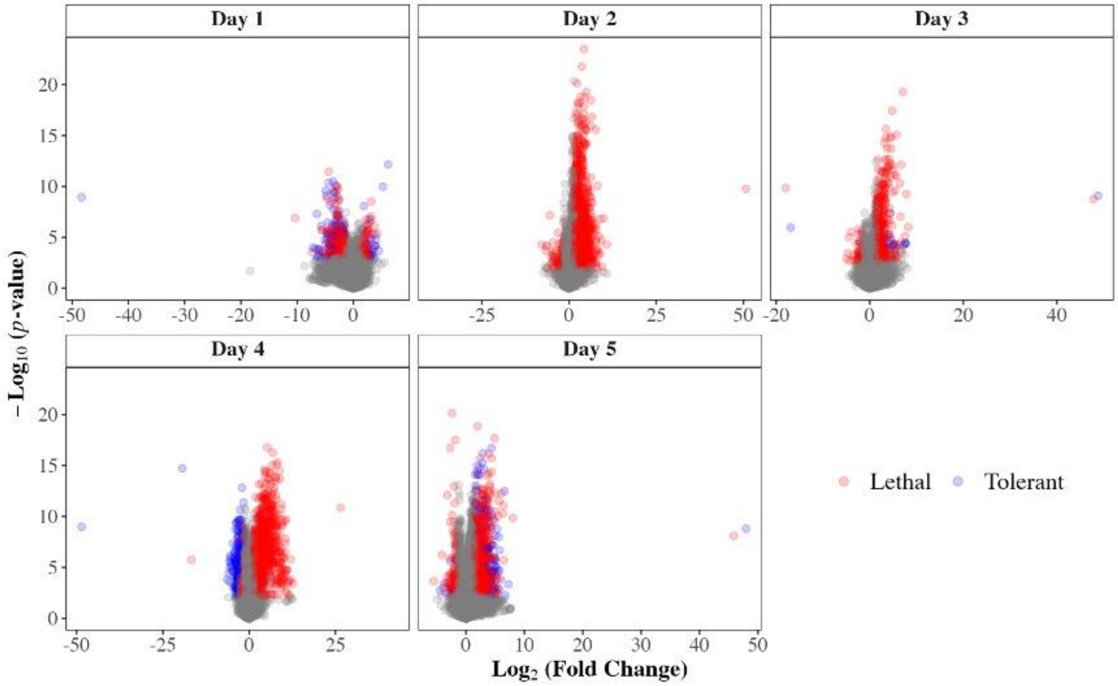

Figure S2. Increased differential gene expression in lethal hDPP4 mice compared to tolerant group. Lung samples were collected on days 1-5 post-infection from hDPP4 mice infected with a tolerant (10^2 $TCID_{50}$) or lethal (10^5 $TCID_{50}$) dose of MERS-CoV. Samples were subjected to RNA-seq followed by data processing and differential expression (DE) analysis relative to time-matched mock-infected controls. The number of DE genes (p<0.05, log2FC > |1.5|) in tolerant and lethal groups were compared. Lung samples were collected from 6 mice per timepoint per dose group.

bioRxiv preprint doi: https://doi.org/10.1101/2025.06.19.660369; this version posted June 19, 2025. The copyright holder for this preprint (which was not certified by peer review) is the author/funder. This article is a US Government work. It is not subject to copyright under 17 USC 105 and is also made available for use under a CC0 license.

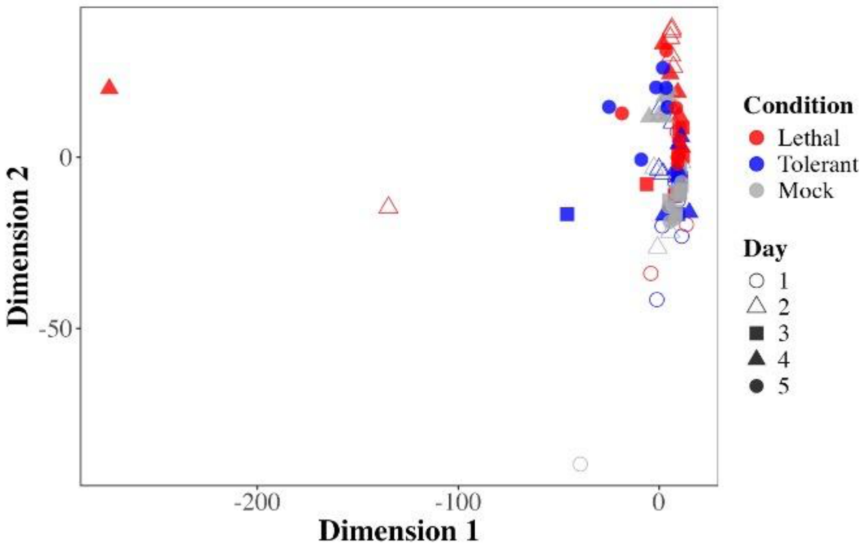

Figure S3. Normalized counts cluster together regardless of dose group. Lung samples from tolerant (10^2 TCID$_{50}$), lethal (10^5 TCID$_{50}$) and mock hDPP4 mice infected with MERS-CoV were collected days 1-5 post-infection and subjected to RNA-seq. Read counts were variance stabilized and subsequently subjected to Euclidean distance calculation to enable multidimensional scaling (MDS). Lung samples were collected from 6 mice per timepoint per dose group.

bioRxiv preprint doi: https://doi.org/10.1101/2025.06.19.660369; this version posted June 19, 2025. The copyright holder for this preprint (which was not certified by peer review) is the author/funder. This article is a US Government work. It is not subject to copyright under 17 USC 105 and is also made available for use under a CC0 license.

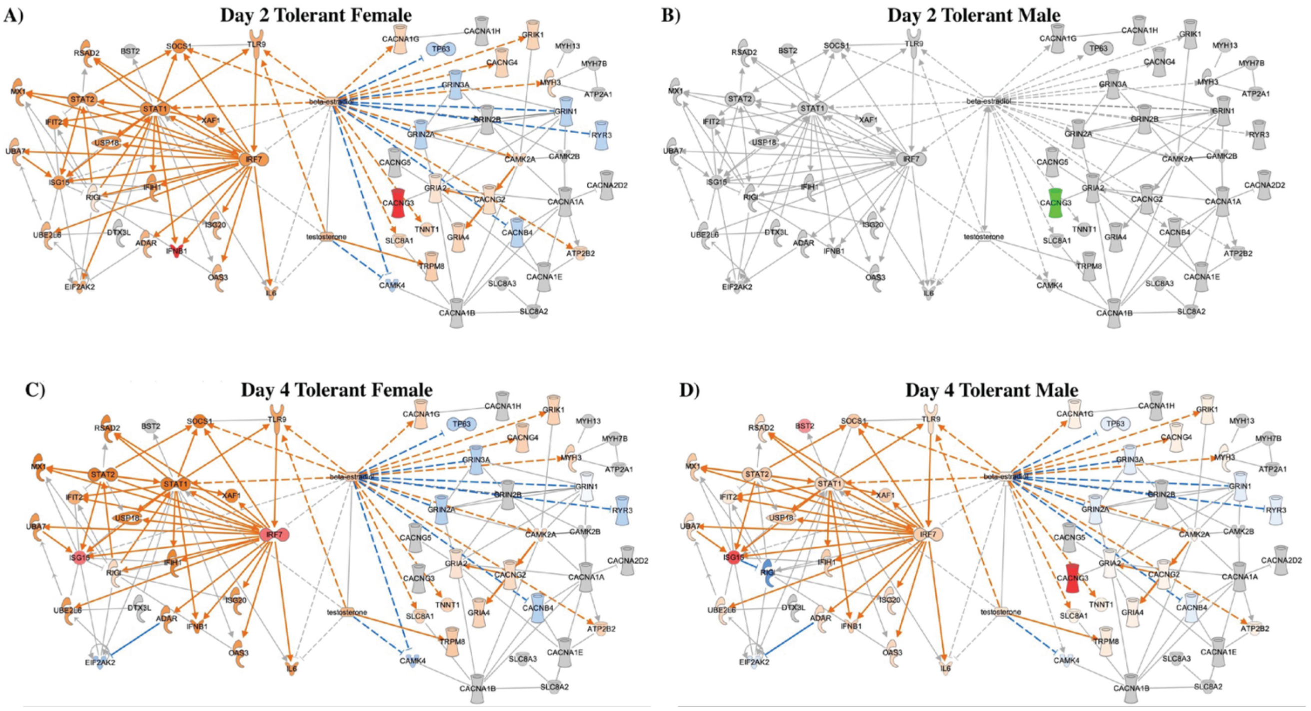

Figure S4. Tolerant female and male hDPP4 mice display temporal differences in their MERS-CoV infection response. Male and female mice were infected with a tolerance-simulating dose of 10^2 $TCID_{50}$ or were mock-infected. RNA samples were collected from the lung on days 1-5 post-infection then underwent RNA-sequencing. Reads were subjected to differential expression (DE) analysis relative to time-matched mock-infected controls. DE genes ($p<0.05$, log2FC > |1.5|) were uploaded to Ingenuity Pathway Analysis (IPA) for custom network mapping (**A**-**D**). DE molecules from antiviral interferon α/β and ISGylation pathways (left) were connected and compared against connected DE molecules from the pro-viral calcium signaling pathway (right). The IPA Overlay tool was used to visualize expression in days 2 and 4 post-infection: red colouring indicated experimentally derived upregulation, while orange and blue represented predicted up or downregulation, respectively. Colour brightness indicated degree of enrichment.

bioRxiv preprint doi: https://doi.org/10.1101/2025.06.19.660369; this version posted June 19, 2025. The copyright holder for this preprint (which was not certified by peer review) is the author/funder. This article is a US Government work. It is not subject to copyright under 17 USC 105 and is also made available for use under a CC0 license.

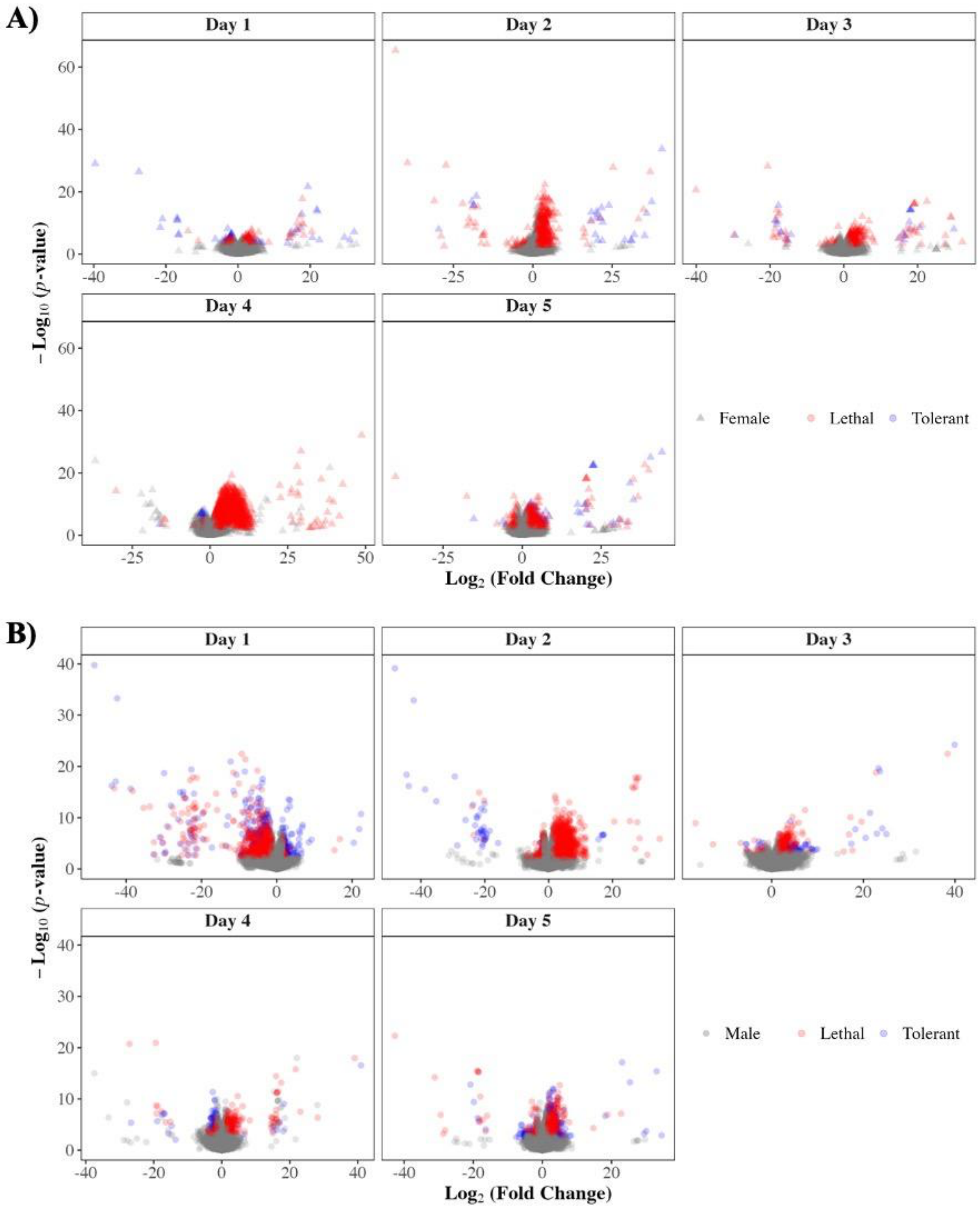

Figure S5. Female and male mice infected with MERS-CoV display dose and sex-dependent differences in DE of genes. hDPP4 mice were infected with a tolerance-inducing (10^2 $TCID_{50}$) or lethal (10^5 $TCID_{50}$) dose of MERS-CoV, or were mock-infected. On days 1-5 post-infection, lung samples were collected and subjected to RNA-seq. Reads underwent differential expression (DE) analysis by comparing lethal or tolerant gene expression to that of

bioRxiv preprint doi: https://doi.org/10.1101/2025.06.19.660369; this version posted June 19, 2025. The copyright holder for this preprint (which was not certified by peer review) is the author/funder. This article is a US Government work. It is not subject to copyright under 17 USC 105 and is also made available for use under a CC0 license.

time-matched mock-infected controls. The number of DE genes (p<0.05, log2FC > |1.5|) in lethal and tolerant groups over time was compared between females (**A**) and males (**B**). Lung samples were collected from 6 mice per timepoint per dose group.

bioRxiv preprint doi: https://doi.org/10.1101/2025.06.19.660369; this version posted June 19, 2025. The copyright holder for this preprint (which was not certified by peer review) is the author/funder. This article is a US Government work. It is not subject to copyright under 17 USC 105 and is also made available for use under a CC0 license.

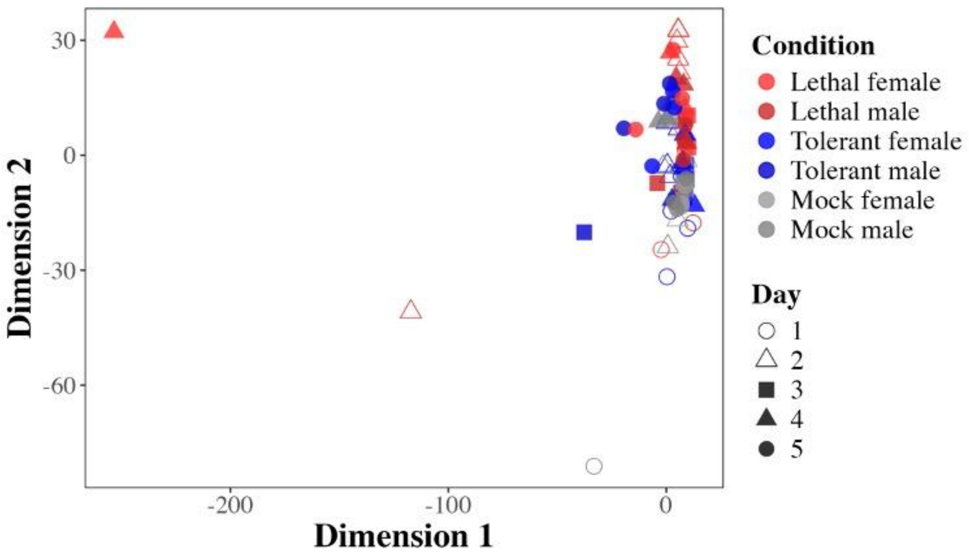

Figure S6. Normalized read counts cluster together regardless of dose group or sex. Lung samples from male and female tolerant (10^2 $TCID_{50}$), lethal (10^5 $TCID_{50}$) and mock hDPP4 mice infected with MERS-CoV were collected days 1-5 post-infection and subjected to RNA-seq. Read counts were variance stabilized and subsequently subjected to Euclidean distance calculation to enable multidimensional scaling (MDS). Lung samples were collected from 6 mice per timepoint per dose group.

bioRxiv preprint doi: https://doi.org/10.1101/2025.06.19.660369; this version posted June 19, 2025. The copyright holder for this preprint (which was not certified by peer review) is the author/funder. This article is a US Government work. It is not subject to copyright under 17 USC 105 and is also made available for use under a CC0 license.

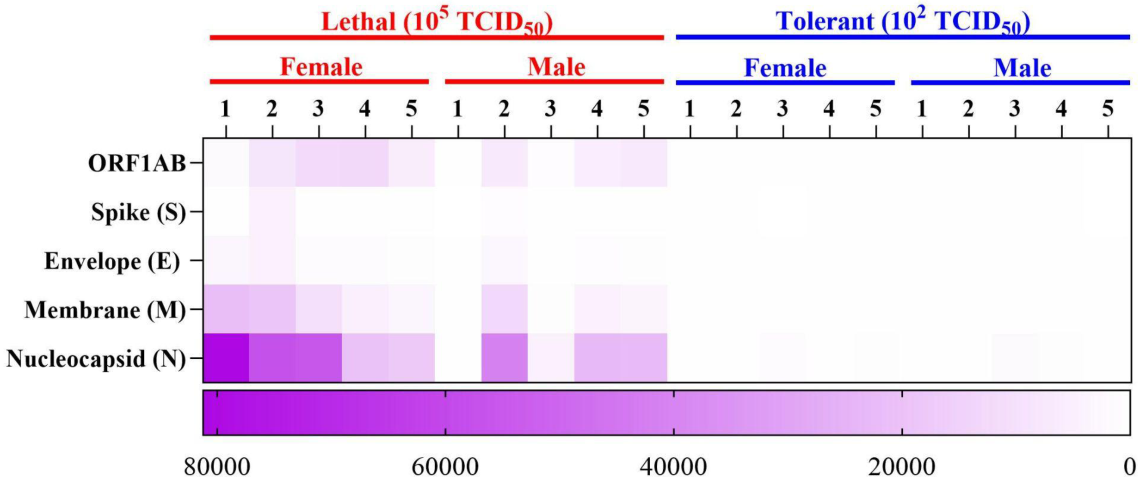

Figure S7. Increased MERS-CoV transcripts in lethal hDPP4 mice compared to tolerant group. Lung samples collected days 1-5 post-infection were collected and subjected to RNA-seq. Reads were processed and filtered to allow alignment of viral transcripts to the MERS-CoV EMC/2012 genome. Read counts across MERS-CoV transcripts were visualized.
